# Correction: Development of a chromium oxide loaded mesoporous silica as an efficient catalyst for carbon dioxide-free production of ethylene oxide

**DOI:** 10.1039/d3ra90116e

**Published:** 2024-01-02

**Authors:** Muhammad Maqbool, Toheed Akhter, Sadaf Ul Hassan, Asif Mahmood, Waheed Al-Masry, Shumaila Razzaque

**Affiliations:** a Department of Chemistry, University of Management and Technology C-II, Johar Town 54770 Lahore Pakistan toheed.akhter@umt.edu.pk; b Department of Chemistry, COMSATS University Islamabad, Lahore Campus Lahore Pakistan; c Department of Chemical Engineering, College of Engineering, King Saud University Riyadh 11421 Saudi Arabia; d Institute of Physical Chemistry, Polish Academy of Sciences Kasprzaka, 44/51 01-224 Warszawa Poland srazzaque@ichf.edu.pl

## Abstract

Correction for ‘Development of a chromium oxide loaded mesoporous silica as an efficient catalyst for carbon dioxide-free production of ethylene oxide’ by Muhammad Maqbool *et al.*, *RSC Adv.*, 2023, **13**, 32424–32432, https://doi.org/10.1039/d3ra05858a.

In the original manuscript, the authors regret errors in the authorship list for ref. 28, with some names being omitted accidently. The corrected reference is shown as ref. 1 below.

The Royal Society of Chemistry apologises for these errors and any consequent inconvenience to authors and readers.

## Supplementary Material
